# Partners in crime: POPX2 phosphatase and its interacting proteins in cancer

**DOI:** 10.1038/s41419-020-03061-0

**Published:** 2020-10-09

**Authors:** Pu Rum Kim, Songjing Zhang, Muhammad Bakhait Rahmat, Cheng-Gee Koh

**Affiliations:** School of Biological Sciences, Nanyang Technological University, Singapore, Singapore

**Keywords:** Metastasis, Cell signalling

## Abstract

Protein phosphorylation and dephosphorylation govern intracellular signal transduction and cellular functions. Kinases and phosphatases are involved in the regulation and development of many diseases such as Alzheimer’s, diabetes, and cancer. While the functions and roles of many kinases, as well as their substrates, are well understood, phosphatases are comparatively less well studied. Recent studies have shown that rather than acting on fewer and more distinct substrates like the kinases, phosphatases can recognize specific phosphorylation sites on many different proteins, making the study of phosphatases and their substrates challenging. One approach to understand the biological functions of phosphatases is through understanding their protein–protein interaction network. POPX2 (Partner of PIX 2; also known as PPM1F or CaMKP) is a serine/threonine phosphatase that belongs to the PP2C family. It has been implicated in cancer cell motility and invasiveness. This review aims to summarize the different binding partners of POPX2 phosphatase and explore the various functions of POPX2 through its interactome in the cell. In particular, we focus on the impact of POPX2 on cancer progression. Acting via its different substrates and interacting proteins, POPX2’s involvement in metastasis is multifaceted and varied according to the stages of metastasis.

## Facts

- POPX2/PPM1F/CaMKP phosphatase has multiple and seemingly opposite roles at different stages of metastasis.
- High POPX2 levels and activity are associated with increased motility and invasiveness of the cancer cells. Low levels and activity favor angiogenesis and establishment of metastatic colonies.
- Similar to other phosphatases, many substrates and interacting proteins of POPX2 have been reported.

## Open questions

- How can POPX2 levels and activity be regulated at different stages of tumorigenesis?
- Is there a switch or switches to control the levels and activity of POPX2 at different phases of tumor development?
- Can we understand the physiological roles of POPX2 or any other protein through exploring its interactome?

## Introduction

Phosphorylation is a posttranslational modification that can modulate the function, subcellular localization, complex formation, and degradation of a protein^1^. Phosphorylation often results in conformational changes of the proteins leading to alterations in their catalytic activities or binding to other proteins. It is a reversible process catalyzed by kinases and phosphatases. Phosphatases specific for dephosphorylating serine/threonine are encoded by two gene families, phosphoprotein phosphatase (PPP) and protein phosphatase Mg^2+^/Mn^2+^-dependent (PPM)^2,3^. PP1, PP2A, and PP2B belong to the PPP family, while PP2C (also known as PPM1) and pyruvate dehydrogenase phosphatase belong to the PPM family. The PPP phosphatases form complexes with other regulatory subunits, whereas the PPM phosphatases exist as monomers and require divalent cations such as Mg^2+^ or Mn^2+^ for activation. The PPM family is insensitive to inhibition by okadaic acid and calyculin A^4,5^. In human, the PPM1 family comprises 12 phosphatases. They are ubiquitously expressed except PPM1E and PPM1J, which are enriched in brain and testis.

**Fig. 1:**
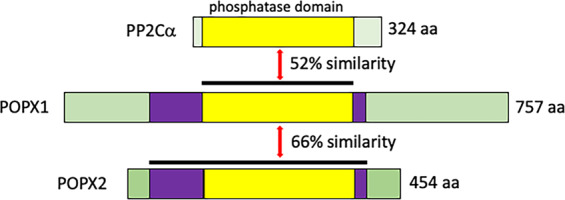
Schematic illustrations of PP2Cα, POPX1, and POPX2. Schematic drawings of the PPM1 phosphatases. POPX1 and POPX2 share high similarity with other PP2C family proteins. The yellow region indicates the PP2C phosphatase catalytic domain and the black bars represent the similarity regions between proteins. The purple region indicates the region of homology between POPX1 and POPX2. The numbers to the right refer to the number of amino acid residues in each protein.

Here, we focus on the PPM1F phosphatase, also known as CaMK phosphatase (CaMKP) or partner of PIX 2 (POPX2), henceforth referred to as POPX2. POPX2 was first discovered in rat brain extract as a dephosphorylation activity against a phospho-peptide containing the Thr286 auto-phosphorylation site of Ca^2+^/calmodulin kinase II (CaMKII)^6^; subsequently, it was found to interact with βPIX and hence was named partner of PIX (POPX)^7^. Partner of PIX 1 (POPX1) and POPX2 are two isoforms of POPX. They share 66% similarity over their core phosphatase domains (Fig. 1). POPX2 localizes in the cytoplasm whereas POPX1 is mainly found in the nucleus^7,8^. The regulation of POPX2 expression and activity by miRNAs has been covered by another review^9^. Here, we attempt to explore the physiological functions of POPX2 through its interactome. In particular, we will focus on POPX2, its partners and their involvement in signaling pathways related to cancer metastasis.

## Exploring the functions of POPX2 through its interactome

Protein–protein interactions can lead to assembly of transient or stable complexes that participate in signaling pathways^10^. Studying the interactome of POPX2 is a useful starting point to explore its cellular functions. Protein interactome can be identified and deduced from immunoprecipitation or pulldown assays, yeast two-hybrid or other similar methods, as well as dry lab approaches and analyses of amino acid sequences, conservation of protein domains throughout evolution and from protein structure-based data^11^. Thus far, POPX2 has been implicated in actin cytoskeleton maintenance, cell motility and invasiveness, angiogenesis, kinesin motor transport, and regulation of apoptosis^7,12–16^. However, not all underlying mechanisms are well understood. In this review, we highlight a number of POPX2 interacting partners identified to date to elucidate the functions of POPX2 in the cells. Since the levels of POPX2 is positively correlated to the motility and invasiveness of the cancer cells^14^, specific emphasis is on understanding the roles of POPX2 in cell migration and metastasis.

## The binding partners of POPX2

Table 1 summarizes known binding partners of POPX2, their cellular roles, and signaling pathways which they are implicated^6,7,12,15,17–23^. The major functions regulated by POPX2 and its binding partners are further discussed below.

**Table 1:** Binding partners of POPX2 and related signaling pathways.

Binding protein	Pathway	Function	Reference
CAMKII	?	Apoptosis	Ishida et al.^6^
βPIX, PAK1	CDC42-βPIX-PAK1	Stress fiber	Koh et al.^7^
mDia1	RhoA-mDia1	Stress fiber	Xie et al.^17^
AMPK	?	Glucose metabolism	Voss et al.^18^
KIF3A	KIF3A-N-Cadherin	Cell adhesion	Phang et al.^19^
KIF3A	KIF3A-N-Cadherin and PAR3/Cdc42	Cell polarity	Hoon et al.^20^
Pcdh-γC5	Pcdh-γC5-CAMKII	Endogenous activator of POPX2	Onouchi et al.^21^
Neurofilament L	?	Neurofilament L-mediated filament assembly	Ozaki et al.^22^
TAK1	TAK1-IKK-NF-kB	Apoptosis	Weng and Koh^15^
LATS1	MST-LATS-YAP/TAZ	Anchorage-dependent growth	Rahmat et al.^12^
Chk1	ATM/ATR-Chk1	Cell-cycle progression	Kim et al.^23^

### Regulation of the actin cytoskeleton and cell motility

Rho GTPases are involved in the regulation of the actin cytoskeleton, which in turn impacts on cell shapes and migration^24–26^. The small GTPases act as molecular switches, which become activated once they are GTP-bound. Many effectors of the Rho GTPases are kinases (e.g., PAK, Rho-associated kinase (ROCK), etc.) and proteins (e.g., mDia1, WASP, etc.), which directly or indirectly influence actin cytoskeleton remodeling in the cells^27^. RhoA, Cdc42, and Rac1 are most well studied amongst the Rho family members. Activated RhoA is associated with increased formation of actin stress fibers^28^, whereas Cdc42 and Rac1 are known to stimulate filopodia and lamellipodia formation, respectively^29,30^. All three GTPases are required for the regulation of cell migration, which involves modulation of actin polymerization and focal adhesion turnover. POPX2 has been found to interact with the effectors and regulators of Rho GTPases.

#### βPIX/PAK

POPX2 was identified as a βPIX interacting protein through a yeast two-hybrid screen^7^. βPIX is a guanine nucleotide exchange factor, which activates Cdc42 and Rac1^31^. POPX2 interacts with βPIX and forms a trimeric complex with βPIX and PAK1. Although βPIX is not a reported substrate of POPX2, POPX2 can dephosphorylate PAK and downregulate its kinase activity. This is an interesting example where an activator and an effector of a Rho GTPase, as well as the negative regulator of the effector are found in the same complex. Active PAK1 induces stress fiber loss and disassembly of focal adhesions^32^. POPX2 overexpression can rescue the loss of stress fibers induced by active PAK1 and dominant negative Cdc42^7^. These observations suggest possible involvement of POPX2 in PAK1 and Cdc42-mediated actin cytoskeleton remodeling. In addition, it has been found that POPX2 could affect the phosphorylation status of myosin light chain (MLC). Silencing POPX2 in MDA-MB-231 cells results in reduced phospho-MLC2^33^. Phosphorylation of MLC by Rho-associated kinase (ROCK) and myosin light chain kinase regulates the activity of myosin II and hence actin–myosin interaction for stress fiber formation and cell contractility^34,35^. Although MLC is not a substrate of POPX2^33^, the phosphatase appears to be able to regulate the stress fibers via a MLC-related mechanism.

#### mDia1

Interestingly, POPX2 also interacts with mDia1, another effector protein of Rho GTPases^17^. mDia1 belongs to the diaphanous family of formins, which catalyzes actin polymerization at the barbed ends of the growing filaments. ROCK and mDia1 are effectors downstream of RhoA that are responsible for the regulation of stress fibers. mDia1 adopts a closed conformation through head-to-tail interaction. This auto-inhibited state can be relieved by binding to active RhoA^36,37^. Expression of a dominant negative form of mDia1, which includes its FH3 domain, leads to breakdown of stress fibers. It has been found that POPX2 interacts with the FH3 domain of mDia1, and overexpression of POPX2 can prevent the loss of stress fibers induced by dominant negative mDia1^17^. Since mDia1 is further implicated in the regulation of serum-response factor (SRF)-mediated transcription, the interaction of POPX2 with mDia1 also impacts transcription mediated by SRF and the transcription co-activator MAL. Although POPX2 interacts with mDia1, it is not clear if mDia1 is a direct substrate of POPX2.

Overexpression of POPX2 leads to robust stress fiber formation and silencing POPX2 results in loss of stress fibers in the cells^7^. These observations and the links between POPX2, PAK, and mDia1 suggest that POPX2 does play a role in stress fiber maintenance which is, in turn, an interplay of activities downstream of Cdc42/Rac1 and RhoA.

### Cell–cell adhesion and cell polarity

Through its interaction with kinesin KIF3 motor complex, POPX2 can affect cell–cell adhesion and cell polarity by inhibiting proper trafficking of cargoes. Known cargoes of the KIF3 motor include N-cadherins, adenomatous polyposis coli, β-catenin, Par-3, fodrin, and Rab11-containing vesicles^38–43^.

#### Kinesin KIF3A

**Fig. 2:**
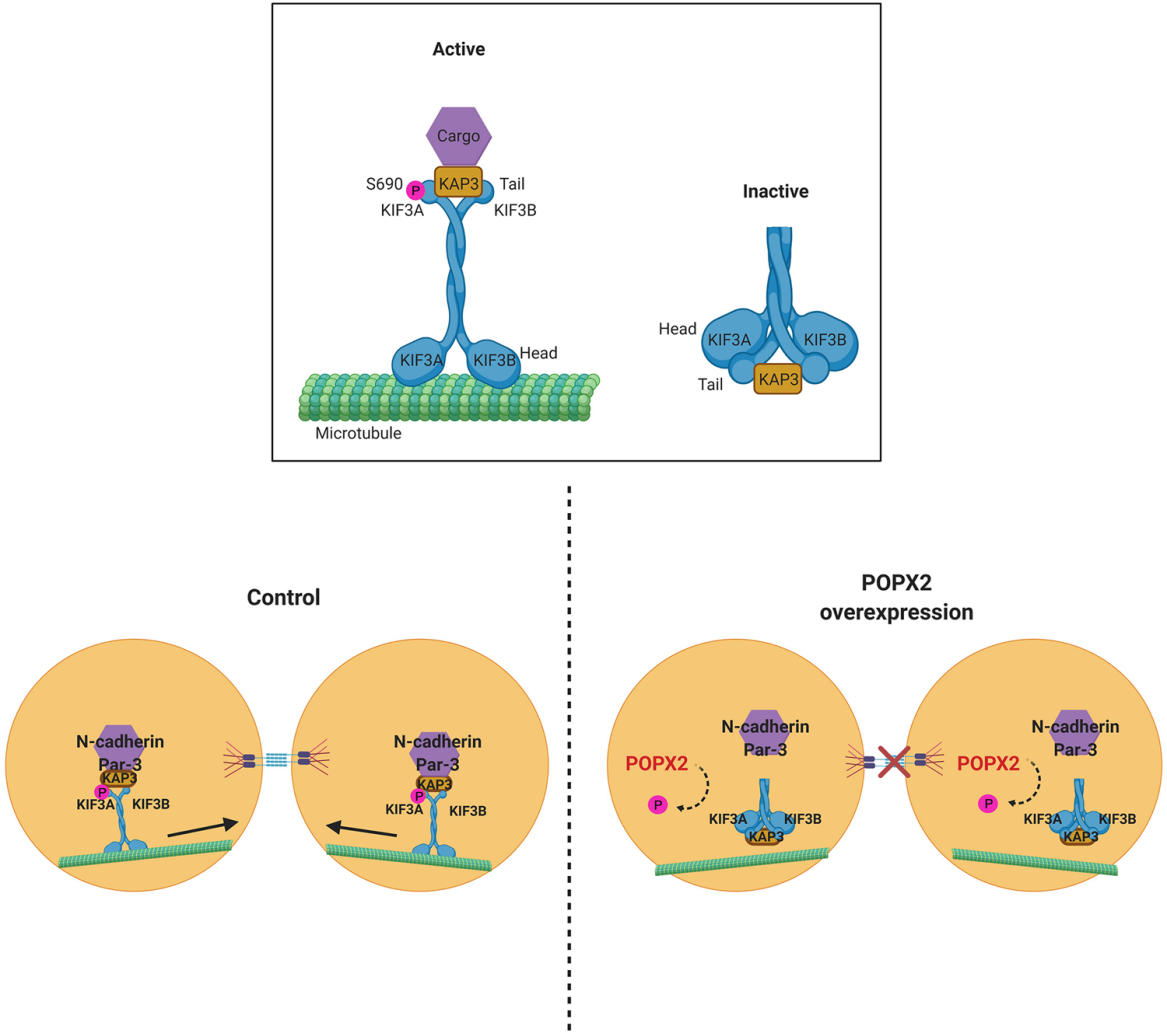
Schematic illustration of KIF3A-mediated cargo delivery in control and POPX2 overexpressing cells. KIF3A motor protein is phosphorylated at Ser690 by CaMKII, leading to an open conformation and active motor. Active kinesin-2 motor complex delivers N-cadherin and Par-3 to the cell surface by trafficking along the microtubules. POPX2 dephosphorylates KIF3A at Ser690 and induces a closed and auto-inhibited conformation. In addition, POPX2 also dephosphorylates and inhibits CaMKII. Therefore, POPX2 overexpressing cells have impaired N-cadherin and Par-3 transport to the cell periphery leading to loss of cell–cell adhesion and cell polarity.

The interaction of POPX2 with the kinesin-2 family motor KIF3A/B complex is an interesting discovery^19^. The KIF3 kinesin motor is a trimeric complex consisting of KIF3A, KIF3B motor subunits and KAP3, the non-motor subunit responsible for cargo binding. POPX2 was first discovered to interact with KAP3 from a yeast two-hybrid screen. Later, KIF3A was also found to interact with POPX2 in overexpression/co-immunoprecipitation experiments. In addition, POPX2 is able to dephosphorylate Ser690 at the C-terminal of KIF3A and affects the trafficking of the motor protein along microtubules. It is proposed that KIF3A adopts an auto-inhibited conformation through head-to-tail interaction when Ser690 is dephosphorylated. As a result, transport of N-cadherin and β-catenin to the cell periphery is impaired in cells overexpressing POPX2, leading to the loss of proper cell–cell contact. Phosphorylation of KIF3A Ser690 by kinases such as CaMKII results in its release from the auto-inhibited state (Fig. 2)^19,44^. It is interesting to note that CaMKII is dephosphorylated by POPX2. Therefore, the effects of POPX2 on KIF3A could also be mediated through the inactivation of CaMKII by POPX2.

In addition to its influence on cell–cell adhesion, POPX2 also regulates cell polarity^20^. Cell polarity is essential for directional migration, differentiation of stem cells, wound healing, and immune response. In migrating fibroblasts, the centrosomes are normally positioned between the leading edges and the nuclei. The nuclear–centrosomal axis and centrosome placement are impaired in POPX2 overexpressing cells thus affecting directional migration. This is most likely due to the loss of proper Par-3 and N-cadherin localization in POPX2 overexpressing cells. Par-3 and N-cadherin are cell polarity regulators that are delivered by kinesin-2 motor to their targeted locations in the cells^45–47^. Defects in Par-3 and N-cadherin transport lead to perturbation of centrosome placement and cell polarity^45^.

### Cell signaling

#### Calcium calmodulin kinases (CaMKs)

POPX2(CaMKP) was first identified and purified as a phosphatase, which dephosphorylated CaMKII in rat brain extract^6^. In the same study, it was found that POPX2 was insensitive to 10-μM okadaic acid and 100-nM calyculin A, two inhibitors which inhibit PP2A and PP1 phosphatases. In addition, POPX2 requires Mn^2+^ but not Mg^2+^ for its activation. Besides CaMKII, POPX2 can also dephosphorylate other CaMKs such as CaMKI and CaMKIV^48^. Using transfected constructs of POPX1/2 homologs from the zebrafish, it has been shown that the phosphatases are capable of dephosphorylating CaMKI in living cells^49^. Since CaMKII is abundant in the brain and controls major neuronal functions, it is expected that POPX2 influences neuronal activities through its regulation of CaMKII. CaMKII is also important for calcium homeostasis^50^. Interestingly, CaMKII has been found to promote breast cancer cell migration, invasion, and anchorage independent growth^51^. CaMKII has also been shown to regulate the cell cycle^52^, which implicates the kinase in the modulation of cell division and proliferation. It is not immediately clear if POPX2 is involved in the regulation of CaMKII in these contexts.

#### Members of the Hippo signaling pathway

**Fig. 3:**
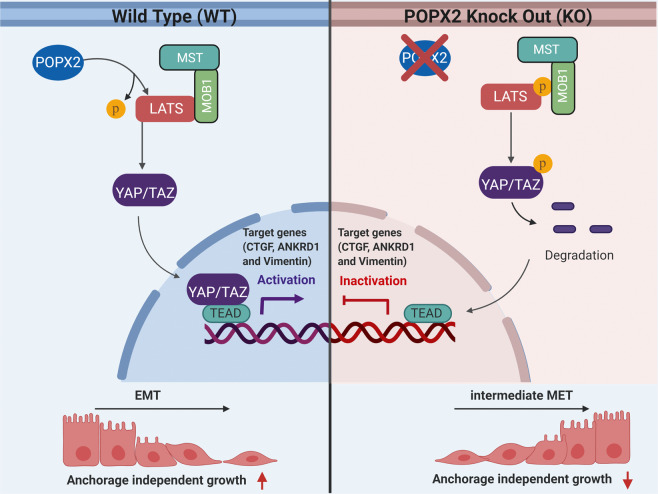
Regulation of the Hippo pathway by POPX2. POPX2 dephosphorylates LATS1 at Thr1079 and negatively regulates the activity of LATS1. In wild-type (WT) cells, dephosphorylation and inactivation of LATS1 result in translocation of YAP/TAZ to the nucleus to induce TEAD-mediated gene transcription. On the other hand, POPX2-knockout (KO) cells show high levels of phospho-LATS1, leading to the phosphorylation of YAP/TAZ. Phosphorylated YAP/TAZ are targeted for degradation in the cytoplasm. As a result, there is decreased TEAD-mediated transcription.

The Hippo pathway is important in controlling organ sizes in animals, and has also been implicated in cancer development^53^. This pathway mainly functions to restrain cell proliferation and promote apoptosis. Cancer cells carrying mutated members of the Hippo pathway acquire anoikis resistance and anchorage independency. The core of the Hippo pathway is made up of a scaffold protein, MOB1, and a kinase cassette consisting of MST1/2 and LATS1/2 kinases. MST phosphorylates LATS and subsequently, active LATS phosphorylates the transcription co-activators, YAP/TAZ^54,55^. Phosphorylated YAP/TAZ then gets sequestered in the cytoplasm via binding to proteins such as 14-3-3 or gets degraded. Non-phosphorylated YAP/TAZ can translocate to the nucleus and interact with transcription factor, TEAD to induce gene expression^56^. Additional kinases such as NDR1/2 have also been found to phosphorylate YAP/TAZ^57^. POPX2 participates in the regulation of the Hippo pathway through binding to core kinases including MST1, LATS1, and NDR1. The interaction between POPX2 and members of the Hippo pathway was first discovered in a POPX2 pulldown/mass-spectrometry study. Later, it was found that POPX2 negatively regulates the activity of LATS1 through dephosphorylating Thr1079^12^. Therefore, cells without POPX2 display higher phosphorylated LATS1 levels and decreased TAZ-target gene expression (Fig. 3).

### Cellular metabolism

#### 5′-AMP-activated protein kinase (AMPK)

5′-Adenosine monophosphate-activated protein kinase (AMPK) consists of α, β, and γ subunits and acts as an energy sensor to regulate cellular energy metabolism through glycogenesis and fatty acid synthesis^58^. AMPK is activated through the binding of AMP to the γ subunit, leading to phosphorylation of Thr172 by liver kinase B1^59^. The interaction between AMPK and POPX1/2 was detected by immunoprecipitation approaches^18^. Depletion of POPX1 and 2 resulted in increased AMPK-Thr172 phosphorylation in HEK293 cells. As activation of AMPK results in reduced glucose production and increased glucose utilization, AMPK has been implicated as a therapeutic target for type 2 diabetes^60^. Therefore, inhibition of POPX1/2 might contribute to type 2 diabetes treatment through activation of AMPK. Recent studies have suggested AMPK as a metabolic tumor suppressor^61^. AMPK is reported to be upstream of mammalian target of rapamycin, cyclooxygenase-2, and nuclear factor erythroid 2-related factor 2 (NRF2), which are associated with cell proliferation, inflammation, and antioxidant response^62^. NRF2 is an antioxidant transcription factor that is phosphorylated by AMPK^62^. Phospho-NRF2 translocates from the cytoplasm to the nucleus, leading to increased antioxidant response element (ARE) gene expression during oxidative stress^63–65^. Although there is no clear evidence showing a direct role for POPX2 in NRF2 activation or phosphorylation status, it is possible that POPX2 activity is inhibited under oxidative conditions, leading to AMPK activation and nuclear accumulation of NRF2 for ARE-mediated gene expression.

### Regulation of apoptosis

#### TGF-β-activated kinase 1 (TAK1)

**Fig. 4:**
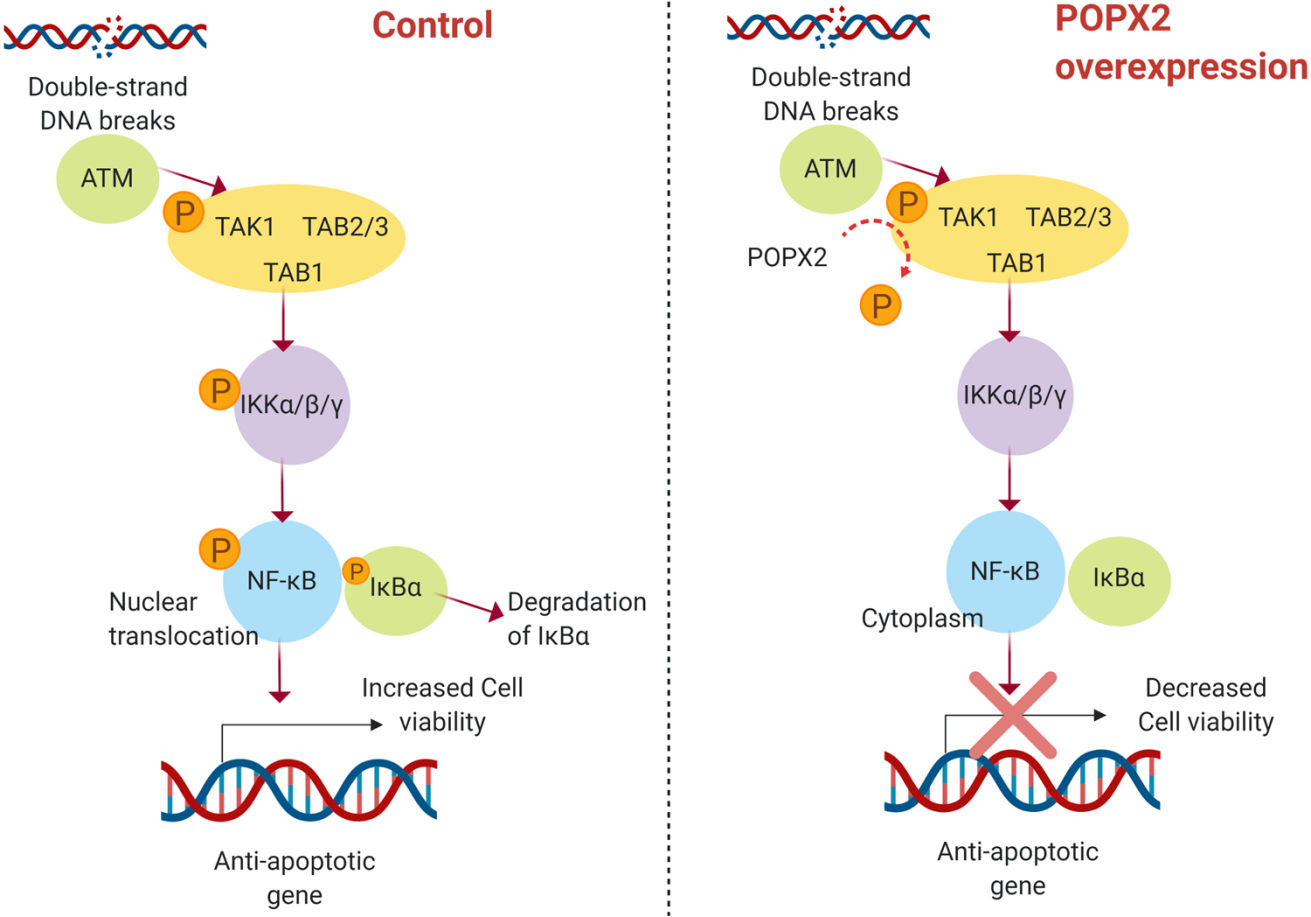
The role of POPX2 in DNA damage response. DNA damage stimulates activation and translocation of ataxia-telangiectasia mutated (ATM) kinase to the cytosol to initiate DNA damage response. TNF-receptor associated factor (TRAF) forms a complex with TAK1 and its binding proteins TAB1/2/3. Active TAK1 phosphorylates the IKK complex, leading to translocation of NF-κB from the cytosol to the nucleus. This results in the upregulation of NF-κB-mediated antiapoptotic gene expression. In the presence of high levels of POPX2, TAK1 is negatively regulated and its downstream effect is inhibited.

TGF-β-activated kinase 1 (TAK1) is important in the regulation of innate and adaptive immunity^66^. It is also a strong pro-survival regulator. TAK1 is activated by cytokines such as interleukin-1 and tumor necrosis factor^67^. The activation of TAK1 requires its interacting protein TAB1 (TAK1-binding protein 1) and TAB2 or TAB3^68–70^. It is well known that TAK1 activates the IKK-NF-κB pathway^71,72^, which is activated in response to genotoxic stress and mediates the balance between anti- and pro-apoptotic gene expression. Binding of TAB1 to the kinase domain of TAK1 promotes auto-phosphorylation of TAK1 at Thr187 in the activation loop^69,73^. Activated TAK1 then phosphorylates IKK causing the release of NF-κB from IκB, leading to NF-κB nuclear translocation thus promoting the expression of antiapoptotic genes^74,75^. TAB1-TAK1 complex is discovered as a binding partner of POPX2 in a FLAG-POPX2 pulldown-mass-spectrometry experiment^15^. Endogenous interaction has been verified by co-immunoprecipitation using whole-cell lysates. POPX2 is found to be able to dephosphorylate TAK1-Thr187 and inactivate TAK1, suggesting that POPX2 can regulate apoptotic gene expression through the modulation of NF-κB activity. It has been shown that in POPX2-knockdown cells, there was increased TAK1 activity and upregulated antiapoptotic gene expression. Therefore, in response to DNA damaging agents, cells with lower POPX2 levels demonstrate higher cell viability possibly through the regulation of the TAK1-IKK-NF-κB pathway (Fig. 4)^15^. An earlier study found that POPX2 showed significant sequence homology and having functional similarity with the FEM-2 gene involved in sex determination in C. elegans^76^. The same study reported that overexpression of POPX2 leds to increased apoptosis in HeLa cells, suggesting that POPX2 might be involved in apoptosis regulation and signaling although the mechanism was not known.

### Cell-cycle regulation

#### Checkpoint kinase 1 (Chk1)

Through a combination of bioinformatics and mass-spectrometry approaches, checkpoint kinase 1 (Chk1) has been identified and verified as a POPX2 interacting protein. Additional co-immunoprecipitation studies of whole-cell lysates further validated the presence of endogenous Chk1-POPX2 complex in the cells. Chk1 is also a potential substrate of POPX2^23^. Chk1 is a serine/threonine kinase involved in cell-cycle regulation. It is crucial for the initiation of the DNA damage checkpoint and impacts on S, G2/M, and M phases of the cell cycle^77–79^. When treated with an etoposide (VP-16), which induces DNA damage, cells expressing high levels of POPX2 accumulate at S phase of the cell cycle. This observation suggests that POPX2 might dephosphorylate Chk1 and inhibit its kinase activity leading to impairment of Chk1-activated G1-S checkpoint, allowing cells to transit from G1 to S phase instead of arresting at G1/S transition^23^.

## The implication of POPX2 in cancer progression

Studies so far have implicated POPX2 in a myriad of signaling pathways and cellular processes including stress fiber maintenance, cargo trafficking via the kinesin motor, apoptosis, glucose metabolism, cell migration, anchorage-dependent growth, and DNA damage response^7,12,14,15,17–20,23,38,80^. Dysregulation of POPX2 will likely lead to development of diseases where these cellular processes and pathways are crucial. POPX2 level is positively correlated to invasiveness of breast cancers^14^. As documented in Oncomine database^81^, higher POPX2 levels are found in triple negative breast cancers (TNBCs) compared to non-TNBCs. POPX2 is also found to enhance cell motility and invasion^14^. Short-term metastasis studies using nude mouse metastasis model found that compared with control MDA-MB-231 breast cancer cells, POPX2-knockdown MDA-MB-231 cells did not colonize and attach well in the lungs, 4 h after tail vein injection^14^. Interestingly, paradoxical results were obtained in longer term metastasis studies using the same model. Although high POPX2 levels were linked to higher cell motility and invasiveness, bigger and more numerous tumor nodules were found in the lungs of nude mice injected with POPX2-knockdown breast cancer cells 8 weeks after injection^16^. It was later confirmed that the secretome of POPX2-knockdown cells contained higher levels of pro-angiogenic factors including cytokines, growth factors, and exosomes. This may explain the bigger tumor nodules formed by the POPX2-knockdown cells.

Cancer metastasis is a complex process containing many stages. It begins with epithelial–mesenchymal transition (EMT), dissemination, and intravasation followed by circulation, extravasation, and colonization^82^. POPX2 might perform different roles at different stages of metastasis. In the following sections, we summarize the implications of POPX2’s involvement at different stages of cancer progression, taking into consideration the various interacting partners of POPX2.

### Epithelial–mesenchymal transition (EMT) (dissemination and migration)

During the process of EMT, tumor cells lose cell adhesion, polarity, and epithelial properties. In the meantime, they acquire migratory and invasive characteristics of mesenchymal cells that enable them to invade into the basement membrane^83^. The proteases that are secreted by the invasive cells degrade the basement membrane and extracellular matrix (ECM), allowing the cells to invade into neighboring tissues.

The screening of POPX2 expression in different breast cancer cell lines found that the level of POPX2 is high in invasive lines such as MDA-MB-231, whereas noninvasive cell lines such as MCF-7 have low POPX2 levels. Depletion of POPX2 in MDA-MB-231 cells significantly reduces cell motility and invasiveness^14,80^, whereas overexpression of POPX2 results in increased cell motility^13^. POPX2 could regulate cell motility through several possible mechanisms. Cells with high POPX2 levels also have high MAPK activity which in turn positively impacts on cell motility^13,80^. Since POPX2 enhances stress fiber formation and maintenance through blocking PAK activity and through a MLC-related mechanism, the phosphatase can thus modulate cell migration through remodeling of the actin cytoskeleton. Meanwhile, POPX2 also interacts with mDia1. This interaction could also lead to changes in actin polymerization. Focal adhesion turnover is also important in cell migration. Through its interaction with βPIX and PAK, POPX2 could potentially regulate focal adhesion turnover. In addition, POPX2 might also exert its influence on focal adhesion through the regulation of the Hippo pathway as YAP has been reported to control focal adhesion assembly^84^. POPX2 can thus participate in the crosstalk between the Hippo pathway and focal adhesion signaling.

The kinase and phosphatase pair, CaMKII and POPX2, regulates KIF3A phosphorylation status and its ability to transport cargoes along the microtubules. The loss of KIF3 cargoes such as N-cadherin at the cell surface leads to impairment of cell–cell adhesion, which in turn can influence the dissemination of tumor cells^19^. Defects in N-cadherin and Par-3 localization also alter centrosome orientation and placement, leading to loss of polarity^20^. Therefore, POPX2 overexpressing cells exhibit reduced cell–cell contacts as well as loss of polarity, which are features of mesenchymal cell^19,20^.

### Tumor cell polarity and intravasation

Metastatic cells are non-polarized and are loosely attached to the ECM for rapid migration^85^. Loss of cell polarity renders the tumor cells more sensitive to external chemotactic signals and become more attracted to signals found in the blood vessels in the primary tumor^86^. POPX2 overexpressing cells showed loss of polarity, which suggests possible roles in EMT^20^. POPX2 also regulates the stability of the microtubule network in migrating cells through the MAPK-stathmin axis^80^. Stathmin promotes microtubule depolymerization and turnover^87–90^. Silencing POPX2 results in decreased MAPK phosphorylation level and its kinase activity, possibly leading to lower phosphorylation of stathmin and changes in microtubule dynamics. Indeed, fewer stable microtubules are observed at the leading edge of POPX2-knockdown cells^80^. Microtubules may not be required for cell migration in most cell types, they are however, required for the maintenance of cell polarity and directed cell migration^91^. In addition, microtubules can influence focal adhesion assembly, maturation, and turnover. Taken together, overexpression of POPX2 in the cells could contribute to tumor invasion through loss of adhesion, polarity and increased cell motility via KIF3A-mediated cargo transport, MAPK pathway activation, and modulation of the microtubules.

PAK is involved in the assembly and disassembly of invadopodia^92,93^. In addition, βPIX is also found to be required for invadopodia formation^94^. Cells with increased βPIX levels show enhanced invadopodia formation. Whether POPX2, as a negative regulator of PAK and an interacting protein of βPIX, has any role in the turnover or regulation of invadopodia is worth investigating.

### Circulation and anoikis resistance

Tumor cells invade into the vasculature and travel to distant locations in the body, where they seed new metastatic colonies^95^. Tumor cells in circulation are deprived of integrin-mediated adhesion to the ECM that is normally essential for cell survival. In physiological context, upon the loss of cell–matrix interactions, epithelial cells undergo anoikis. However, circulating tumor cells (CTCs) acquire anoikis resistance, thus allowing them to survive in circulation^96^. POPX2 has recently been shown to promote anoikis resistance by suppressing LATS1-mediated Hippo pathway. High levels of POPX2 in the cells reduce the activity of LATS1, leading to increased YAP/TAZ nuclear translocation. This, in turn, leads to TEAD-mediated gene expression and the upregulation of proliferation- and anchorage independence-related gene expression^12^. Therefore, it is likely that high levels of POPX2 in the cancer cells can upregulate anchorage independence, leading to survival of tumor cells without cell–matrix interactions.

### Metastatic colonization and angiogenesis

Metastasis is the most-deadly phase of tumor progression to the cancer patient. The CTCs extravasate from blood vessels and attach to the parenchyma of distant tissues. The tumor cells are able to traverse the endothelial wall of the blood capillaries through a process called trans-endothelial migration (TEM)^97^. TEM and vascular integrity are influenced by secreted proteins such as vascular endothelial growth factor and matrix metalloproteinases. These secreted proteins are important for promoting intravasation and extravasation during metastasis^97,98^. In order for the tumor cells to thrive in the new microenvironment of the distant tissues, they require stimulation by additional secreted proteins such as growth factors and cytokines. Secretome study using the conditioned media from POPX2-knockdown breast cancer cells demonstrated that silencing POPX2 promotes the secretion of cytokines and growth factors^16^. Consistently, in vitro angiogenesis assays showed that the conditioned media collected from POPX2-knockdown cells increases tube length and vessel branch points compared to those from control cells^16^. Furthermore, it was also found that the conditioned media from POPX2-knockdown cells contains higher amounts of exosomes^16^, which could also contribute to angiogenesis^99^. The secretome study of POPX2-knockdown cells highlighted the paradoxical roles of POPX2 at different stages of cancer.

POPX2 acts as a multifaceted regulator of cancer metastasis. The phosphatase promotes dissemination and migration of tumor cells to blood vessels and survival of cells during circulation through the CaMKII-KIF3A pathway, MAPK pathway, and the Hippo pathway, whereas POPX2 also inhibits cancer metastasis by reducing cytokines secretion and angiogenesis. This is further supported by data from cancer patient samples in Oncomine. Analysis using the cancer microarray database shows that POPX2 gene expression is high in invasive TNBC compared with non-TNBC, while POPX2 gene expression is low at metastatic sites compared with primary cancer sites in many different types of cancers^16,81^.

## The function of POPX2 in cancer treatment and resistance

Drug resistance invariably limits the efficacy of cancer therapies. Cancer cells acquire resistance to chemotherapies by many different mechanisms. The cancer cells can increase drug efflux, enhance DNA damage repair, activate alternative pathways to circumvent the inhibition of the drug targets, and undergo mutation of the drug targets^100–102^. The DNA damage pathway is an attractive drug target because its activation is associated with resistance to genotoxic therapies^103^. Interestingly, POPX2 has been implicated in the regulation of cell sensitivity toward an etoposide (VP-16), which induces DNA damage. POPX2 could promote apoptosis through inactivation of TAK1 kinase in response to DNA damaging drug^15^. Therefore, it is likely that low levels of POPX2 can favor inhibition of apoptosis in response to DNA damage, resulting in increased cell survival.

Recent findings of POPX2 and Chk1 interaction^23^ shed light on how POPX2 might modulate the phosphorylation status of Chk1 and influence cell-cycle progression. Active Chk1 initiates the DNA damage checkpoint that results in arrest at the G1-S phase of the cell cycle. When there is DNA damage, cells with higher levels of POPX2 appear to be able to proceed to S phase compared to those with low levels, which are arrested at G1/S transition due to the activation of Chk1-regulated checkpoint. Interestingly, it has recently been shown that Chk1 activity is oncogenic and is required for the development of blood cancer and lymphoma^104^. Higher levels of Chk1 in acute myeloid leukemia patients allow the cancer cells to tolerate higher degree of DNA damage^105^. Therefore, targeting Chk1 or its upstream kinases could be an option to overcome chemoresistance^106^.

## The regulation of POPX2 activity

**Fig. 5:**
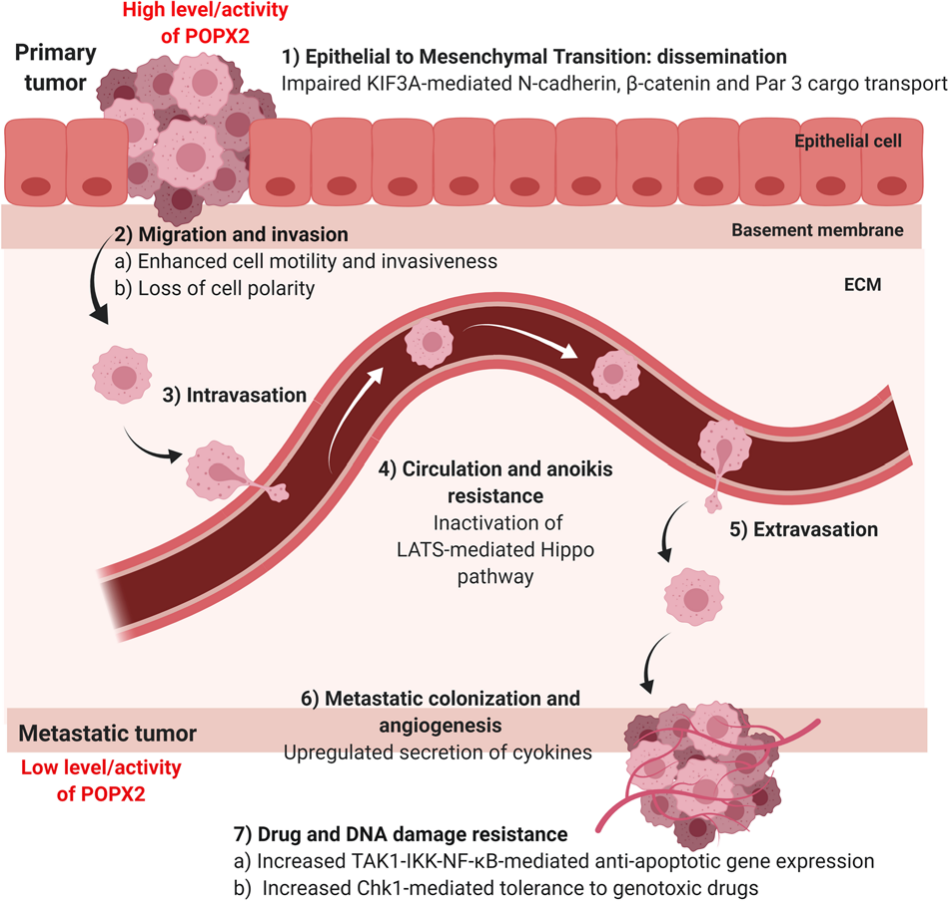
Proposed working model of POPX2 in cancer progression. High levels or activity of POPX2 at early stages of metastasis favor cancer cell invasion, migration, and survival in circulation (stages 1, 2, and 4). However, lower levels or activity of POPX2 in cancer cells which have established at metastatic sites promote colonization through upregulation of pro-angiogenic factors and secreted proteins (stage 6). Meanwhile, lower levels or activity of POPX2 also enhance TAK1-mediated antiapoptotic gene expression, which in turn allows the cancer cells to proliferate despite suffering DNA damage (stage 7).

POPX2 appears to have different roles in cancer progression (Fig. 5). The level and activity of the phosphatase vary at different stages of metastasis. Higher levels of POPX2 favor the initial EMT, whereas lower levels appear to be beneficial for establishing metastatic colonies. It is possible that POPX2 levels and activity are actively regulated in cancer cells. Investigating the activators and inhibitors of POPX2 would be crucial to study its physiological function and clinical application. To date, there are only a few studies concerning the regulators of POPX2. Since PPM phosphatases are activated by Mn^2+^ or Mg^2+^, it was found that POPX2 can also be activated by polycations such as poly-L-lysine^107^. Protocadherin (Pcdh)-γC5 has been identified as an endogenous regulator of POPX2. CaMKI, POPX2, and the C-terminal fragment of Pcdh-γC5 form a ternary complex, which allows Pcdh-γC5 to enhance the activity of POPX2 to dephosphorylate CaMKI^21^. POPX2 activity can be inhibited by hydrogen peroxide, while the presence of reducing agent can restore POPX2 activity^108^. The oxidation/reduction status of Cys359 of POPX2 is identified to be crucial in the regulation of its activity. Disulfide bond formation of Cys359 with other cysteine residues (Cys259/Cys315) might lead to conformational changes that result in an inactive phosphatase. These findings suggest that reactive oxygen species (ROS) present in the cells could potentially affect the activities of POPX2. Moreover, high levels of ROS are present in cancer cells due to increased proliferation and metabolic activities^109^. Although high levels of ROS promote oncogenic phenotypes and tumor progression, excessive levels of ROS will lead to cell-cycle arrest, apoptosis, and senescence. Certain chemotherapeutic drugs are designed to reduce the antioxidant levels in tumor cells, which can then lead to cell death^110^. Could ROS function as a switch to control POPX2 activity at different stages of tumor development? This is an interesting question. Besides, it has been suggested that a redox imbalance may exist between primary tumor cells and the more aggressive metastatic cells. Sequential changes of redox balance could influence gene expression and protein stability, thus driving cancer progression^111^. It might be possible that redox balance tunes POPX2 activity and stability at different tumor stages, which in turn, modulates cancer progression.

Sueyoshi et al. have reported that Evans Blue and Chicago Sky Blue 6B, which are potent inhibitors of vesicular uptake of L-glutamate, inhibit POPX2^112,113^. However, these compounds also inhibit other enzymes, suggesting that they are not selective inhibitors of POPX2. The naphthol derivative, 1-amino-8-naphthol-2, 4-disulfonic acid, has been shown to selectively inhibits the activity of POPX2 on CaMKI and the activity of POPX1 on CaMKIV^113^. There has been no further development of these inhibitors for potential clinical uses. Besides chemical inhibitors of POPXs, microRNAs might be regulating POPX2 expression throughout tumor progression. POPX2 has been reported to be a direct substrate of miR-200c in breast cancer cells. Silencing POPX2 phenocopies miR-200c-mediated inhibition of migration and invasion, suggesting POPX2 might be downstream of miR-200c in regulating cell migration and actin re-organization^33^. Luo et al. has reported that miR-149 negatively regulates POPX2 expression levels in hepatocellular carcinoma. Overexpression of POPX2 could partially reverse miR-149-mediated inhibition on invasion and migration^114^. Gene silencing involving tRNase Z^L^ and small noncoding RNA has also been reported to target POPX2^115^. These noncoding RNAs may function to regulate the levels of POPX2 differently in healthy or cancer cells. More work is required to determine if these noncoding RNAs can function as a “switch” to control POPX2 levels at different stages of tumor development.

The balance between phosphorylation and dephosphorylation is finely modulated by kinases and phosphatases. Dysregulation of these posttranslational modifications can lead to defects in signal transduction and other biological processes that contribute to cancer development. Therefore, investigating potent inhibitors and activators of POPX2 could have important implication for anticancer therapy.

## Conclusion

The in vivo functions of POPX2 can be better understood through the discovery and validation of its interacting proteins. Investigations thus far have suggested that POPX2 plays important roles in regulating cell migration and invasion, cell polarity, apoptosis, and cell-cycle checkpoint control. Since POPX2 has been found to interact with many different proteins, it is not surprising that POPX2 can regulate a variety of cellular processes implicated in tumorigenesis. Further studies on how POPX2’s activities and levels are regulated will help to better understand the function of POPX2 at different stages of tumorigenesis. More work is required to identify the specific contexts in which activation or inhibition of POPX2 may be applied toward therapeutic intervention of cancer.
